# Calendar

**DOI:** 10.1155/S1463924683000152

**Published:** 1983


Journal of Automatic Chemistry, Volume 5, Number 1 (January–March 1983), pages 47–48

### Calendar

### Editor's note

*Organizers of conferences, seminars, etc. should send details for inclusion in the calendar as soon as the relevant information is available and not later than three months before the event.*

### March 1983

**34th Pittsburgh Conference and Exposition on Analytical Chemistry and Applied Spectroscopy**
To be held from 7 to 12 March in Atlantic City, New Jersey, USA.
*Registration forms from Peter Castle, Registration Chairman, PO Box 10590, Pittsburgh, Pennsylvania 15325, USA.*

**Problems in Process Analysis**
Organized by the Royal Society of Chemistry's Automatic Methods Group and the Industrial Analytical Instrumentation Group of the Institute of Measurement and Control. To be held on 8 March at the Bloomsbury Centre Hotel, London.
*Further information from Dr C. J. Jackson, Occupational Hygiene Laboratory, 403 Edgware Road, London NW2 6LN.*

**Microcomputers in Analytical Chemistry**
Northern Ireland Region: Royal Society of Chemistry. To be held on 15 March at Queen's University, Belfast, UK.
*Further information from W. J. Swindall, Department of Chemistry, David Keir Building, Queen's University, Belfast BT9 5AG, UK.*

**185th American Chemical Society National Meeting**
To be held from 20 to 25 March in Seattle, Washington, USA.
*Further information from A. T. Winstead, American Chemical Society, 1155 16th Street, N.W., Washington, D.C. 20036.*

### April 1983

**International Symposium on Electroanalysis in Biomedical, Environmental and Industrial Sciences**
Organized by the Electroanalytical Group and Western Region of the Analytical Division of the Royal Society of Chemistry. To be held from 5–8 April at UWIST, Cardiff, UK.
*Further information from the Short Courses Section, UWIST, King Edward VII Avenue, Cardiff CF1 3NU, UK.*

**Royal Society of Chemistry Annual Chemical Congress**
To be held from 11 to 13 April at the University of Lancaster, UK.
*Further information from Dr John F. Gibson, Royal Society of Chemistry, Burlington House, London W1V 0BN.*

**Advanced Analytical Concepts for the Clinical Laboratory**
To be held from 21 to 23 April in Gatlinburg, Tennessee, USA.
*Further information from Carl A. Burtis, Chemical Technology Division, Oak Ridge National Laboratory, PO Box X, Oak Ridge, Tennessee 37830, USA.*

**Kongress für Laboratoriumsmedizin**
To be held from 19 to 23 April in Vienna, Austria.
*Further information from Interconvention, Postfach 80, A 1107, Vienna, Austria.*

**Fourteenth Annual Pittsburgh Conference on Modelling and Simulation**
To be held from 21 to 22 April in Pittsburgh, Pennsylvania, USA.
*Further information from William G. Vogt or Marlin H. Mickle, Modelling and Simulation Conference, 348 Benedum Engineering Hall, University of Pittsburgh, Pittsburgh, Pennsylvania 15261, USA.*

**1983 Conference on Artificial Intelligence**
To be held from 26 to 27 April in Rochester, Michigan, USA.
*Further information from Professor Nan K. Loh, Center for Robotics and Advanced Automation, School of Engineering, Oakland University, Rochester, Michigan 48063, USA.*

### May 1983

**VIIth International Symposium on Column Liquid Chromatography**
To be held from 2 to 6 May in Baden-Baden, FR Germany.
*Further information from Gesellschaft Deutscher Chemiker, Postfach 90 04 40, Varrentrappstrasse 40, D 6000 Frankfurt 90, FR Germany.*

**Biotechnology for Fuels and Chemicals: Fifth Oak Ridge National Laboratory Symposium**
To be held from 10 to 13 May in Gatlinburg, Tennessee, USA.
*Further information from Charles D. Scott, Chemical Technology Division, Oak Ridge National Laboratory, PO Box X, Oak Ridge, Tennessee 37830, USA.*

**Haemonetics Research Laboratory: Tenth International Advanced Apheresis Seminar**
To be held from 16 to 17 May in Boston, Massachusetts, USA.
*Further information from Douglas A. Moe, Haemonetics Research Institute, 400 Wood Road, Braintree, Massachussetts 02184, USA.*

### June 1983

**Tetronica 83**
To be held at Earls Court in London from 14–17 June 1983.
*Further information from Industrial & Trade Fairs Ltd, Radcliffe House, Blenheim Court, Solihull, West Midlands B91 2BG, UK.*

**Modern Methods of Food Analysis**
A basic science symposium organized by the Institute of Food Technologists. To be held from 17–18 June in New Orleans, USA.
*Further information from either Dr John Whitaker, University of California, Davis, California 95616, USA; or Dr Kent K. Stewart, Department of Food Science & Technology, Virginia Polytechnic Institute and State University, Blacksburg, Virginia 24061, USA.*

**International Symposium on Chromatography and Mass Spectrometry in Nutrition Science and Food Safety**
To be held from 20 to 22 June in Montreux, Switzerland.
*Further information from the Italian Group for Mass Spectrometry in Biochemistry and Medicine, Via Eritrea 62, I 20157 Milan, Italy.*

**ISA at Transducer/Tempcon 83**
The Instrument Society of America is organizing a two-day event at the Transducer/Tempcon exhibition and conference at the Wembley Conference Centre, London, from 28–30 June. The event will be subject to separate registration.
*Further information from Norma Thewlis,*

*Trident International Exhibitions Ltd, 21 Plymouth Road, Tavistok, Devon PL19 8AU, UK.*

### July 1983

**5th European Congress of Clinical Chemistry**
To be held from 3 to 8 July in Budapest, Hungary.
*Further information from MOTESZ Congress Bureau, PO Box 32, H 1361 Budapest, Hungary.*

**Computers in Automation and Laboratory Management**
The fifth Summer School of Automatic Chemical Analysis. The course is residential and organized by Professor J. Betteridge, D. Porter and Dr P. Stockwell. To be held from 10 to 15 July at the University of Sussex, UK.
*Further information from Beverly Humphrey, 176a North View Road, London N8 7NB.*

**SAC 83: International Conference and Exhibition on Analytical Chemistry**
Organized by the Analytical Division of the Royal Society of Chemistry. To be held from 17–23 July at the University of Edinburgh.
*Further information from Miss P. E. Hutchinson, Analytical Division, Royal Society of Chemistry, Burlington House, London W1V 0BN.*

### August 1983

**Computing in Clinical Laboratories**
To be held from 24 to 26 August in Breda, The Netherlands.
*Further information from Mr R. C. J. Galle, Stichting Medische Laboratoria, Bergschot 69, 4817 PA Breda, The Netherlands.*

### October 1983

**Analyticon 83**
To be held from 12 to 14 October at the Barbican Centre, London.
*Further information from Mr G. C. Young, SIMA (Scientific Instrument Manufacturers' Association of Great Britain), Leicester House, 8 Leicester Street, London WC2H 7BN.*

### 1984 meetings

**XIIth International Congress of Clinical Chemistry**
To be held from 29 April to 4 May 1984 in Rio de Janeiro, Brazil.
*Further information from the Executive Secretary, XIIth International Congress, Rua Vicente Licinio 95, Cep 20270, Rio de Janeiro, Brazil.*

**2nd International Congress on Automation and New Technology in the Clinical Laboratory**
To be held from 15 to 18 October 1984 in Barcelona, Spain.
*For further information contact 2nd International Congress on Automation and New Technology in the Clinical Laboratory, IV Congreso Nacional de la Sociedad Española de Química Clínica, Apartado de Correos 543, Barcelona, Spain.*

**14th Annual Symposium on the Analytical Chemistry of Pollutants and the 3rd International Congress on Analytical Techniques in Environmental Chemistry**
To be held from 22–24 November 1984 at the Palacio de Congresos in Barcelona, Spain.
*For further information contact 14th Annual Symposium—3rd International Congress—Expoquimia, Avda. Reina Mª. Christina, Barcelona 4, Spain.*

PLASMA-THERM

The Market Leader for ICP Sources and Accessories

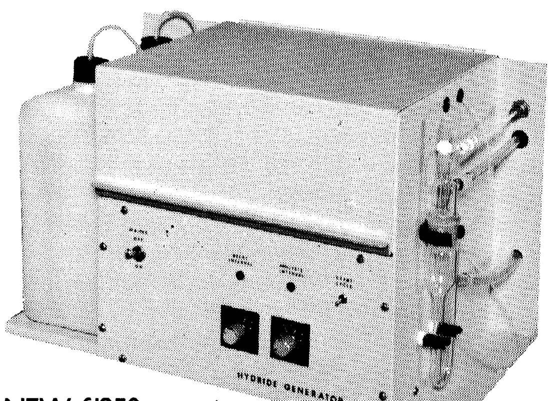

NEW £1950 per unit

Automated Hydride Generator

Rapid analysis of As, Bi, Sn, Sb, Se, Te, Ge, Hg, & Pb. Detection levels in region of .1 PPB for above elements. Stable continuous hydride generation allows greater precision and reliability. Simple visual checking of instrument performance. Sample levels measured against background. Easy and simple to interface to any plasma or atomic absorption instrument. Additional pumping channel available for introduction of $H_2O_2$ or Iodide solution. Stand-alone or remote operation from computer or auto sampler.

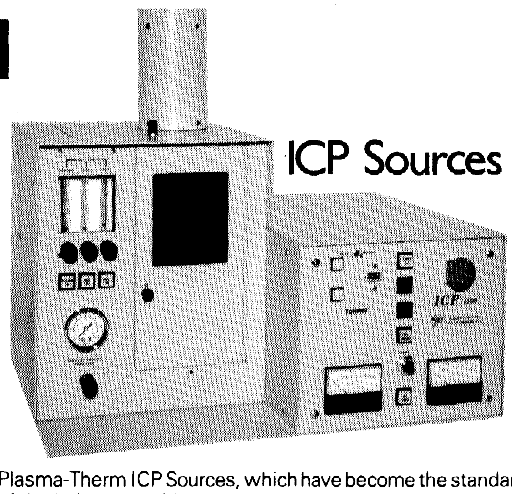

ICP Sources

Plasma-Therm ICP Sources, which have become the standard of the industry are ideal sources for macro or trace, single or multielement analysis. The heart of each system is a crystal controlled RF generator (27.12 or 40.68 MHz) designed for stability, reliability, serviceability, simplified control and safety. Automatic power control; impedance matching; and precise flow meter control of plasma, auxiliary, and nebulizing gases ensure high spectroanalytical stability and reproducibility. All systems are easily adapted to most commercial spectrometers.

The recently introduced Compact Single Phase 40 MHz Systems available as 1KW and 2KW versions offer increased stability, reliability and performance.

PLASMA-THERM LTD

2/3 Kangley Bridge Road Lower Sydenham SE26 5AR Tel: 01-778 6798

Circle No. 5 on Reader Enquiry Card

